# Ethanolic extract of *Schizonepeta tenuifolia* attenuates osteoclast formation and activation in vitro and protects against lipopolysaccharide-induced bone loss in vivo

**DOI:** 10.1186/s12906-016-1300-0

**Published:** 2016-08-22

**Authors:** Ju-Young Kim, Jong Min Baek, Sung-Jun Ahn, Yoon-Hee Cheon, Sun-Hyang Park, Miyoung Yang, Min Kyu Choi, Jaemin Oh

**Affiliations:** 1 Imaging Science-based Lung and Bone Diseases Research Center, Wonkwang University, Iksan, Jeonbuk 570-749 Republic of Korea; 2 Institute for Skeletal Disease, Wonkwang University, Iksan, Jeonbuk 570-749 Republic of Korea; 3 Institute for Environmental Science, Wonkwang University, Iksan, Jeonbuk 570-749 Republic of Korea; 4 Department of Anatomy, School of Medicine, Wonkwang University, 344-2 Sinyong-dong, Iksan, Jeonbuk 570-749 Republic of Korea

**Keywords:** *Schizonepeta tenuifolia*, Osteoclast differentiation, Bone resorption, Osteoporosis

## Abstract

**Background:**

Excessive osteoclast activity is a major cause of metabolic bone disorders, such as osteopenia, rheumatoid arthritis, and osteoporosis. Thus, discovery of agents targeting osteoclast differentiation and bone resorption is important for development of novel treatments for bone diseases. It has been demonstrated that ethanolic extract of *schizonepeta tenuifolia* (EEST) has potent anti-oxidant and anti-inflammatory activities. However, the beneficial effects of EEST on bone metabolism have not been studied. Therefore, we intend to investigate the effects of EEST on osteoclast differentiation.

**Methods:**

We examined the effects and mechanisms of action of the EEST on osteoclastogenesis in vitro in bone marrow macrophages (BMMs) stimulated with receptor activator of nuclear factor kappa-B ligand (RANKL) and in vivo using a mouse model of lipopolysaccharide (LPS)-induced bone destruction.

**Results:**

We found that EEST inhibited phosphorylation of Akt and IkB at early stages of RANKL-induced osteoclastogenesis. Furthermore, EEST negatively controlled the transcription and translation levels of nuclear factor of activated T cells c1 (NFATc1) and the translation level of c-Fos at the final stage of osteoclast differentiation. Reflecting these effects, EEST blocked both filamentous actin (F-actin) ring formation and bone resorbing activity of mature osteoclasts in vitro. The inhibitory effects of EEST on osteoclast formation and activity were observed in an LPS-mediated bone erosion mouse model using micro-CT and histological analysis.

**Conclusions:**

EEST is a potential agent that is able to treat osteoclast-related bone diseases, such as osteoporosis.

## Background

Skeletal tissue continuously undergoes remodeling. This is defined by three physiological processes. First, in the resorption phase, osteoclasts dissolve the old bone. Next, in the reversal phase, mononuclear cells arrive on the bone surface to complete resorption stage. Finally, in the formation phase, osteoblasts initiate formation of new bone matrix in response to signals of the mononuclear cells [1]. Osteoclast over-activity, caused by such risk factors as an inflammatory response and estrogen hormone deficiency, expedites perturbation of steady-state bone remodeling. This leads to severe bone diseases, including osteoporosis and osteopenia, which are directly associated with excessive bone destruction and impaired bone quality [2, 3].

The initiation of osteoclast differentiation from hematopoietic stem cell of the monocyte/macrophage lineage is dependent on stimulation by two important cytokines: macrophage colony-stimulating factor (M-CSF), which is required for osteoclast proliferation and survival, and receptor activator of nuclear factor kappa-B ligand (RANKL), which triggers various signals for osteoclastogenesis by binding to the RANK receptor, the surface marker of osteoclast precursors [4, 5]. In response to M-CSF and RANKL stimulation, tumor necrosis factor receptor-associated factor6 (TRAF6) is recruited, which leads to subsequent activation of downstream transducers of the RANKL-dependent pathway. The downstream transducers include mitogen-activated protein kinases (MAPKs), such as p38, c-Jun N-terminal kinase (JNK), and extracellular signal-regulated kinase (ERK); Akt; and nuclear factor kappa-B (NFκB). Activation of this signaling pathways leads to the nuclear translocation of c-Fos and nuclear factor of activated T cell c1 (NFATc1), which are recognized as two master osteoclast regulators. This results increase expression of various osteoclast-specific marker genes that are crucial for development and function of mature osteoclasts, such as *β3-integrin*, *dendritic cell-specific transmembrane protein* (*DC-STAMP*), and *Cathepsin K* [6–10].

Lipopolysaccharide (LPS) leads to the intracellular induction of p38, JNK, and NFκB in macrophages and monocytes, and promotes the differentiation and survival of osteoclasts through the production of other factors such as PGE_2_, interleukin 1, RANKL, and TNF [11–13]. Therefore, LPS is an important mediator of pathological bone destruction associated with inflammation.

In this study, we screened several plant-derived extracts by tartrate-resistant acid phosphate (TRAP) staining and confirmed that ethanolic extract of *Schizonepeta tenuifolia* (EEST) can suppress osteoclast activity. Although previous reports demonstrated that EEST exerts various pharmacological effects, including anti-inflammatory, anti-oxidant, and hemostatic activity, the effects of EEST on bone metabolism have not been studied [14–16]. Therefore, we investigated the effects of EEST on RANKL-induced osteoclast differentiation and its underlying intracellular mechanisms in vitro. Furthermore, we performed in vivo experiments using a LPS-mediated bone erosion mouse model in order to verify the therapeutic value of EEST for treatment of osteoporosis.

## Methods

### Plant materials and EEST preparation

The 95 % EEST (sale number: CA03-094) of the Korean Plant Extract Bank (KPEB) at the Korea Research Institute of Bioscience and Biotechnology (KRIBB) (Daejeon, Korea) was acquired from the plant samples purchased from an Oriental medicine market in Korea and then authenticated by three taxonomic experts at Chungbuk, Chungnam, and Pusan National University. Also, all forms of extraction from the KPEB were produced through standardization procedure. The KPEB extraction protocol consists of 5 stages: extraction, filtration and yield testing, concentration, drying, and storage. First, extraction of ST was performed using 95 % ethanol with a sonicator (SDN-900H, SD Ultrasonic Cleaner, Seoul, Korea) at 45 °C for 3 days (15 min sonication followed by 2 h standing; repeated 10 times per day). Next, The EEST was filtered through Whatman filter paper No.2 (Advantec, Tokyo, Japan). The filtrates were combined, evaporated under vacuum, and then lyophilized with a CleanVac 12 vacuum freeze dryer (Biotron; Gangneung, Korea) at -70 °C for 24 h under reduced pressure (<20 Pa). A 50 mg/mL stock solution of EEST was prepared in dimethyl sulfoxide (DMSO) and stored at -20 °C.

### Reagents

A TRAP staining solution was obtained from Sigma Aldrich (St. Louis, MO, USA) and a sodium 3ʹ-[1-(phenyl-aminocarbonyl)-3,4-tetrazolium]-bis(4-methoxy-6-nitro) (XTT) assay kit was purchased from Roche (Indianapolis, IN, USA). The α-minimum essential medium (α-MEM), fetal bovine serum (FBS), and penicillin-streptomycin were purchased from Gibco-BRL (Grand Island, NY, USA), and soluble human recombinant M-CSF and RANKL were purchased from Peprotech (London, UK). Specific antibodies against c-Fos and NFATc1 were obtained from Santa Cruz Biotechnology (Santa Cruz, CA, USA). Specific primary antibodies against phospho-p38, p38, phospho-Akt, Akt, phospho-ERK, ERK, phospho-JNK, JNK, phospho-IκB, and IκB were purchased from Cell Signaling Technology (Beverly, MA, USA), and that against the house-keeping gene GAPDH was purchased from Santa Cruz Biotechnology.

### Osteoclast differentiation from mouse bone marrow macrophages (BMMs)

To obtain osteoclast precursors, we prepared mouse BMMs as described previously [17] and BMMs were incubated with M-CSF (30 ng/mL) and RANKL (50 ng/mL) in the absence and presence of EEST (1–50 μg/mL). In this experiment, the control group was treated with 0.1 % DMSO, and the other 5 groups were treated with EEST at concentrations of 1, 5, 10, 25, and 50 μg/mL. After 3 days, the culture medium was replaced with fresh medium with the same composition. After an additional day, cells were stained with a TRAP solution and TRAP-positive multinucleated cells (TRAP^+^ MNCs) containing more than 5 nuclei were observed and counted as described previously [17].

### Evaluation of cytotoxicity, analysis of western blotting and quantitative real-time reverse transcriptase polymerase chain reaction (RT-PCR), retroviral gene transfection, and assay of bone resorption in co-culture system of BMCs and primary osteoblasts

The experiments were performed as described previously [17]. The primer sets used for the real-time PCR were listed in Table 1. The retroviral vectors used for gene transfection were pMX-IRES-EGFP, pMX-Akt-IRES-EGFP, and pMX-constitutively active (CA)-IKKβ-IRES-EGFP packaging.

**Table 1:** Primer sequences used for real-time PCR analysis

Gene name		Primer sequence (5′ → 3′)
*GAPDH*	Forward	5′-TCA AGA AGG TGG TGA AGC AG-3′
	Reverse	5′-AGT GGG AGT TGC TGT TGA AGT-3′
*c-Fos*	Forward	5′-GGT GAA GAC CGT GTC AGG AG-3′
	Reverse	5′-TAT TCC GTT CCC TTC GGA TT-3′
*NFATc1*	Forward	5′-GAG TAC ACC TTC CAG CAC CTT-3′
	Reverse	5′-TAT GAT GTC GGG GAA AGA GA-3′
*Cathepsin K*	Forward	5′-CCA GTG GGA GCT ATG GAA GA-3′
	Reverse	5′-CTC CAG GTT ATG GGC AGA GA-3′
*β3-integrin*	Forward	5′-GGA GTG GCT GAT CCA GAT GT-3′
	Reverse	5′-TCT GAC CAT CTT CCC TGT CC-3′
*Atp6v0d2*	Forward	5ʹ-GAC CCT GTG GCA CTT TTT GT-3ʹ
	Reverse	5ʹ-GTG TTT GAG CTT GGG GAG AA-3ʹ
*DC-STAMP*	Forward	5′-TCC TCC ATG AAC AAA CAG TTC CA-3′
	Reverse	5′-AGA CGT GGT TTA GGA ATG CAG CTC-3′

### Immunofluorescence staining and confocal microscopy

BMMs were incubated with M-CSF (30 ng/mL) and RANKL (50 ng/mL) in the presence and absence of EEST (50 μg/mL). After 3 days, the culture medium was replaced with fresh medium containing the same constituents. After an additional day, cells were stained with phalloidin and a DAPI solution for visualization of filamentous actin (F-actin) and nuclei as described previously [17]. The fluorescence signal was observed using a laser scanning confocal microscope (Olympus FV1200; Olympus, Shinjuku, Japan), and images representative of 5 experiments were analyzed using Image-Pro Plus software (Media Cybernetics Inc., Rockville, MD, USA).

### LPS-mediated bone erosion mouse model and micro-CT and histological analysis

Five-week-old male ICR mice were purchased from Samtako Inc. (Osan, Korea). The mice were kept under controlled temperature (22–24 °C) and humidity (55–60 %) with a 12 h light/dark cycle. The use of experimental animals was reviewed by the institutional animal care and use committee (IACUC) and approved under WKU15-91. To examine the effect of EEST on LPS-induced bone destruction, 5-week-old male ICR mice were randomly divided into 3 groups (5 mice per group): Control (treated with PBS), LPS (treated only with LPS), and LPS + ST (treated with both LPS and EEST). EEST (200 mg/kg) or PBS was administered orally 1 day before LPS injection (5 mg/kg), and then every other 8 days. LPS was injected intraperitoneally on day 2 and 6. All mice were sacrificed after 8 days and femur of each mouse were examined by high-resolution micro-CT analysis and histological analysis including hematoxylin and eosin (H&E) and TRAP staining as described previously [17]. Briefly, micro-CT analysis was performed using bone-related parameters, including bone volume fraction (BV/TV), trabecular thickness (Tb.Th), trabecular separation (Tb.Sp), and trabecular number (Tb.N) which are minimal set of variables that should be investigated for trabecular bone regions [18]. Nomenclature, symbols, and units used in this study were recommended by the American Society for Bone Mineral Research (ASBMR) Nomenclature Committee.

### Statistical analysis

Each experiment was conducted at least 3 times, and data were expressed as mean ± standard deviation (SD). All statistical analyses were performed using Statistical Package for the Social Sciences Software (SPSS; Korean version 14.0). Student’s *t*-test was used to compare parameters between 2 groups, while analysis of variance followed by Tukey post-hoc test was used to compare parameters among 3 groups. *P* < 0.05 was considered statistically significant.

## Results and discussion

### EEST exerts inhibitory effects on TRAP-positive osteoclast formation induced by RANKL treatment without cytotoxicity

**Fig. 1:**
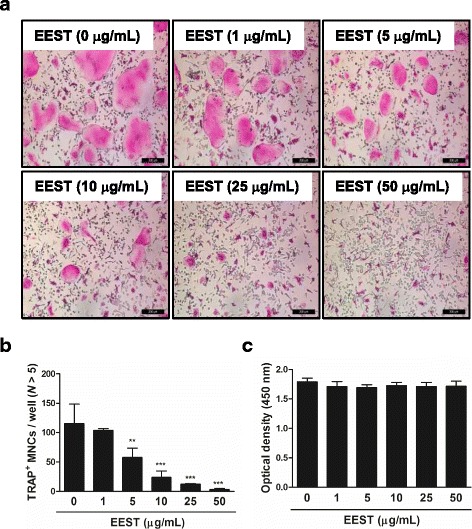
EEST attenuates RANKL-induced osteoclast differentiation in a dose-dependent manner with no cytotoxicity. **a** BMMs were cultured for 4 days in the presence of M-CSF (30 ng/mL) and RANKL (50 ng/mL) with the indicated concentrations of EEST. Cells were fixed in 3.7 % formalin, permeabilized with 0.1 % Triton X-100, and stained with TRAP solution. TRAP^+^ MNCs were photographed under a light microscope. **b** TRAP^+^ MNCs with more than 5 nuclei were counted. ***P < 0.001, **P < 0.01 *vs*. control. **c** BMMs were seeded into a 96-well plate and cultured for 3 days in the presence of M-CSF (30 ng/mL) and the indicated concentrations of EEST. After 3 days, the absorbance at 450 nm was determined using an ELISA reader

To screen the effects of EEST on osteoclast formation, we treated mouse BMMs with the indicated concentrations of EEST in culture medium (α-MEM containing 30 ng/mL M-CSF and 50 ng/mL RANKL). We observed that EEST suppressed the number of TRAP^+^ MNCs with more than 5 nuclei in a dose-dependent manner compared to DMSO-treated control group (Fig. 1a and b). In addition, EEST did not exert any cytotoxic effects during the differentiation of BMMs into osteoclasts (Fig. 1c). Our results indicated that EEST effectively suppressed RANKL-dependent osteoclast formation without cytotoxicity at various concentrations (1–50 μg/mL).

### EEST affects early signaling events of osteoclastogenesis *via* dephosphorylation of Akt and IkB

**Fig. 2:**
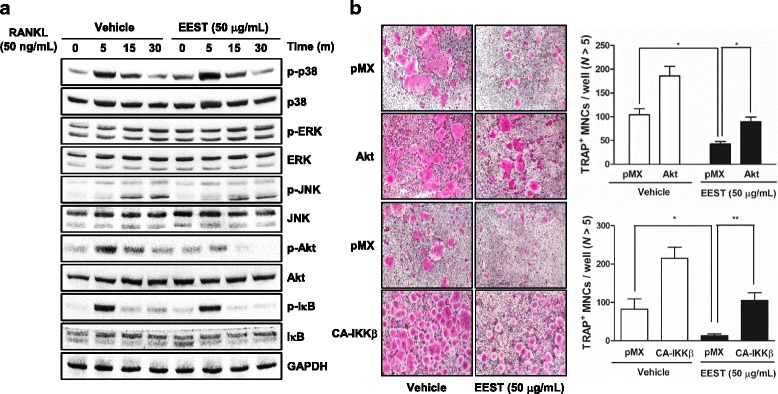
EEST down-regulates RANKL-mediated phosphorylation of Akt and IkB. **a** BMMs were cultured for 1 day in the presence of M-CSF (10 ng/mL). Next, BMMs were starved for 3 h, pretreated with EEST (50 μg/mL) for 1 h and then stimulated with RANKL (50 ng/mL) for the indicated times. Cell lysates were analyzed by western blotting with antibodies against p-p38, p38, p-ERK, ERK, p-JNK, JNK, p-Akt, Akt, p-IκB, IκB, and GAPDH. **b** BMMs were infected with retroviruses expressing pMX-IRES-EGFP (pMX), pMX- CA-IKKβ-EGFP, and pMX-Akt-EGFP. Infected BMMs were cultured with or without EEST (50 μg/mL) in the presence of M-CSF (30 ng/mL) and RANKL (50 ng/mL) for 4 days. After culturing, the cells were fixed and stained for TRAP (*left*). The TRAP+ MNCs with more than 5 nuclei were counted (*right*). *P < 0.05, **P < 0.01 *vs*. the indicated group

Next, we performed western blotting to confirm whether EEST was related with RANKL-dependent signal transducers, such as MAPKs, including p38, ERK, and JNK; Akt; and IκB. As shown in Fig. 2a, EEST strongly reduced the phosphorylation of Akt and weakly suppressed the phosphorylation of IkB. In addition, we reaffirmed the role of EEST on the activation of Akt and IκB using retroviral vectors. Overexpression of Akt and CA form of IKKβ, a catalytic subunit of IκB kinase complex, was sufficient to reverse the inhibitory effect of EEST on osteoclast formation. Previously, it has been shown that Akt plays a critical role in osteoclast survival by regulating its downstream target, GSK3β, and the signaling cascade of NFATc1, a master transcription factor for osteoclastogenesis. An Akt inhibitor, LY294002, significantly suppresses osteoclast formation and NFATc1 expression in vitro, and systemic injection of LY294002 attenuates multiple myeloma-induced abnormal osteoclast formation and osteolysis in vivo [19, 20]. The other early signaling molecule, IκB, is also essential for the regulation of osteoclast activity. The inhibitor of IκB kinase (IKK), which induces phosphorylation of IkB, suppresses RANKL-induced osteoclast formation and activity in vitro, and ovariectomy-mediated bone erosion in vivo by targeting osteoclastic bone resorption [21]. Therefore, our results suggested that EEST suppresses osteoclast formation by targeting two signal transducers, Akt and IκB at the early stage of osteoclast differentiation.

### EEST suppresses mRNA and protein expressions of NFATc1 and protein expression of c-Fos

**Fig. 3:**
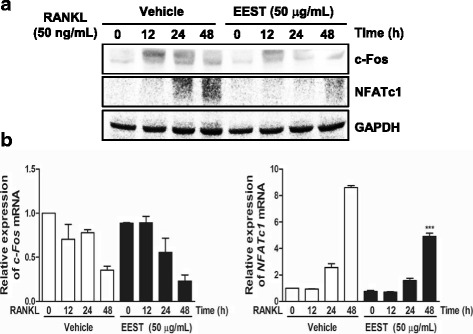
EEST inhibits protein expression of c-Fos and both mRNA and protein expression of NFATc1. **a** BMMs were pretreated with or without EEST (50 μg/mL) for 1 h and then stimulated with M-CSF (30 ng/mL) and RANKL (50 ng/mL) for the indicated times. The cell lysates were analyzed by western blotting with antibodies against c-Fos, NFATc1, and GAPDH. **b** BMMs were stimulated with RANKL (50 ng/mL) and M-CSF (30 ng/mL) in the presence or absence of EEST (50 μg/mL) for the indicated times. Total RNA was isolated from cells using the QIAzol reagent, and mRNA expression of c-Fos and NFATc1 was determined using quantitative real-time RT-PCR. ***P < 0.001 *vs*. control in the indicated time

Through the verification of the role of EEST on the early stage of osteoclastogenesis, we assumed that EEST was also associated with the final stage of RANKL-mediated osteoclast differentiation. At this stage, the transcription factors c-Fos and NFATc1 are activated in response to the activation of early signal transducers [22, 23]. It has previously been shown that embryonic stem cells with NFATc1 deficiency are not capable of differentiating into functional osteoclasts, and the retrovirus-mediated overexpression of NFATc1 induces normal osteoclast differentiation even in the absence of RANKL [22]. Ectopic expression of c-Fos reverses the osteoclast dysfunction-induced symptom of osteopetrosis, which is observed in c-Fos knockout mice [23]. In this study, we found that EEST blocked the expression of NFATc1 gene at both the transcription and translation levels and c-Fos gene at the translation level (Fig. 3). Collectively, EEST showed suppression effects on the expression of two major transcription factors in response to downregulation of Akt and IκB during osteoclast differentiation.

### EEST inhibits formation of F-actin structure and bone resorption activity of mature osteoclasts

**Fig. 4:**
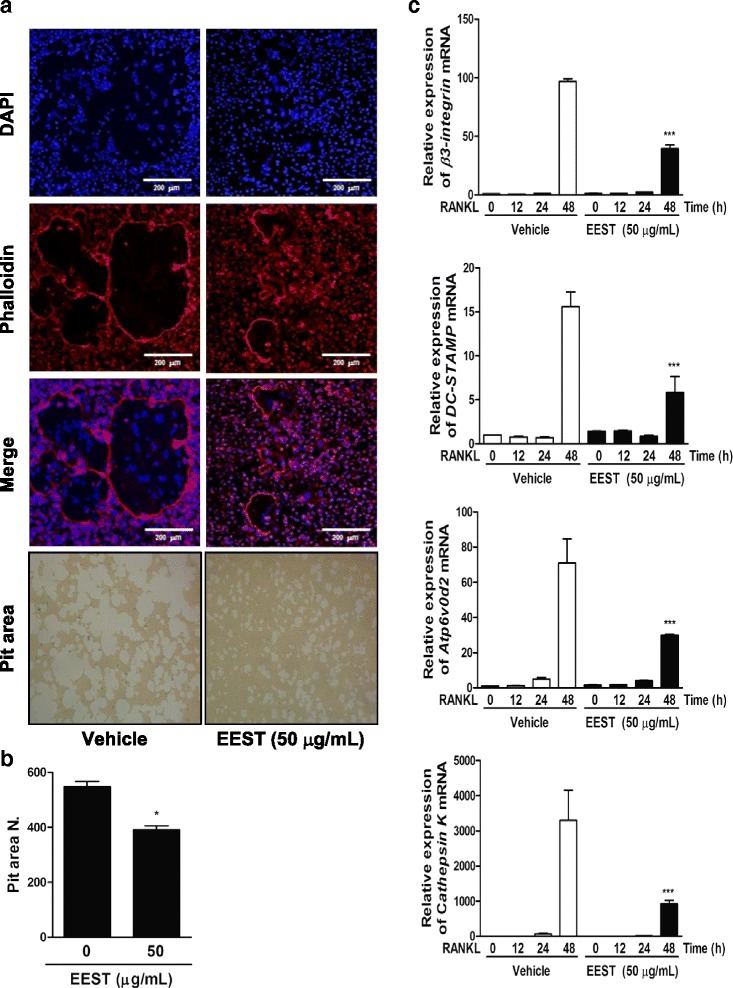
EEST negatively controls F-actin structure, bone-resorbing activity, and expression of osteoclast-specific genes. **a** BMMs were cultured for 4 days in the presence of M-CSF (30 ng/mL) and RANKL (50 ng/mL) with or without EEST (50 μg/mL). Cells were fixed with 3.7 % formalin, permeabilized with 0.1 % Triton X-100, and stained with phalloidin and DAPI. Mature osteoclasts were seeded on hydroxyapatite-coated plates for 24 h with EEST (50 μg/mL) treatment. Adherent cells were removed and photographed under a light microscope. **b** The relative ratio of pit areas was quantified using Image J. ***P < 0.001 *vs*. control. **c** BMMs were stimulated with RANKL (50 ng/mL) and M-CSF (30 ng/mL) in the presence or absence of EEST (50 μg/mL) for the indicated times. Total RNA was isolated from cells using QIAzol reagent, and mRNA expression of *β3-integrin*, *DC-STAMP*, *Atp6v0d2*, and *Cathepsin K* was determined using quantitative real-time RT-PCR. ***P < 0.001 *vs*. control in the indicated time

During the development of functional osteoclasts, the organization of F-actin structure is required for bone resorption activity. Once the multinucleated osteoclast attaches to the bone surface, its membrane becomes polarized and secretes both hydrogen ions and lytic enzymes into the resorption lacuna in order to dissolve the bone matrix. This region is surrounded by a tight sealing zone that is recognized as a physiological feature of mature osteoclasts and composed of F-actin ring-like structures [24, 25]. In this study, we confirmed that EEST significantly inhibited the formation F-actin ring-positive osteoclasts and subsequently reduced the pit area formed as a result of the bone resorption activity of mature osteoclasts (Fig. 4a and b). Also, this phenomenon was induced by the decreased expression of various osteoclast-marker genes, including *β3-integrin*, *DC-STAMP*, *Atp6v0d2*, and *Cathepsin K*, which are required for the cell-to-cell fusion needed to organize F-actin structure and bone resorption [26–29]. Our results demonstrated that EEST negatively regulated the development of functional osteoclasts by attenuating the transcription of several osteoclast-specific marker genes.

### EEST exerts a protective effects in LPS-induced bone erosion mice model

**Fig. 5:**
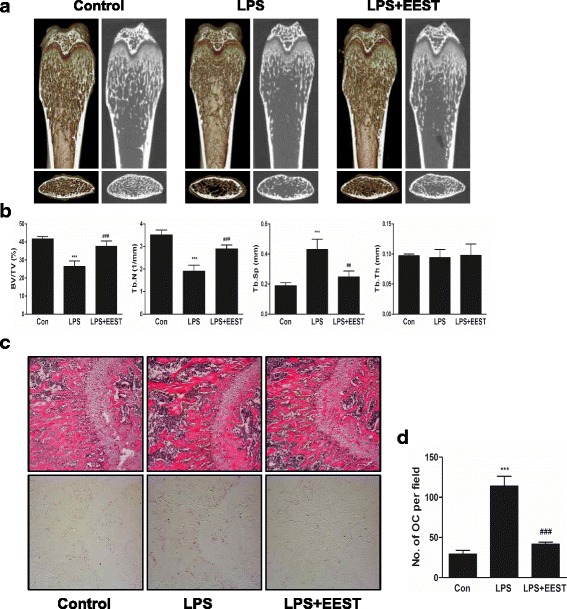
EEST recovers LPS-induced inflammatory bone loss in mice. **a** Mice were sacrificed 8 days after the first LPS injection and 2D or 3D radiographs of the coronal and transverse planes of the proximal femora were obtained by micro-CT. **b** The BV/TV, Tb.Sp, Tb.Th, and Tb.N of the femora were determined using the micro-CT data and analyzed by INFINITT-Xelis software. ***P < 0.001 *vs*. control; ^##^ *P* < 0.01, ^###^ *P* < 0.001 *vs*. LPS group. **c** Dissected femora were fixed, decalcified, embedded, and sectioned. Sections were stained with TRAP and H&E. **d** The number of osteoclasts per field of tissue was counted by histomorphometric analysis

Finally, to determine if the in vitro effect of EEST on osteoclast activity could be confirmed in vivo, commonly used mouse model of osteoporosis was applied. Among the osteoporotic models, in this study, we focused on the therapeutic value of EEST on inflammation-induced bone loss because EEST has been proved to exert a significant anti-inflammatory effect [14]. LPS is an efficient tool for the induction of inflammatory osteoporosis in mice [30]. Thus, we selected bone loss model induced by LPS injection to suggest the therapeutic value of EEST on inflammatory osteoporosis. Mice were injected with LPS intraperitoneally on day 1 and 4 to induce systemic inflammatory response and subsequent bone loss and orally administered with EEST or PBS every 8 days. After, the left femora of sacrificed mice were analyzed by micro-CT and the right femora were stained with H&E and TRAP solution. While the bone mass in the femora of LPS-treated mice was lower than that of controls, there was a partial recovery of bone density in mice treated with both LPS and EEST (Fig. 5a). Morphometric analysis of the femora of LPS-treated mice showed decreased BV/TV and Tb.N and increased Tb.Sp, while restoration of these parameters was observed in the LPS + EEST group (Fig. 5b). Histological analysis confirmed that LPS + EEST treatment inhibited LPS-induced erosion of bone matrix and formation of TRAP-positive osteoclasts within growth plates (Fig. 5c and d). The present findings suggested that EEST exhibited protective effects on osteoclast differentiation and subsequent bone resorption in vivo. Also, it was thought that our further study could be in need of demonstrating the effect of EEST on OVX-mediated bone loss to more clarify the protective effect of EEST on osteoporosis.

## Conclusions

In the present study, we demonstrated that EEST attenuated RANKL-induced osteoclast differentiation by downregulating Akt and IkB phosphorylation in the early signaling event and subsequently targeted NFATc1 at the transcriptional and translational levels and c-Fos at the translational level. Moreover, EEST exerted suppression effects on F-actin ring formation and bone resorption in vitro and LPS-mediated bone erosion in vivo. Taken together, our findings supported the potential value of EEST as a plant-derived therapeutic agent to treat bone-related disorders, particularly osteoporosis.

## Abbreviations

BMCs: Bone marrow cells
BMMs: Bone marrow macrophages
BV/TV: Bone volume per tissue volume
DC-STAMP: Dendritic cell-specific transmembrane protein
EEST: Ethanolic extract of *Schizonepeta tenuifolia*
ELISA: Enzyme-linked immunosorbent assay
ERK: Extracellular signal-regulated kinase
F-actin: Filamentous actin
FBS: Fetal bovine serum
GAPDH: Glyceraldehyde 3-phosphate dehydrogenase
H&E: Hematoxylin and eosin
IKK: Inhibitor of IkB kinase
JNK: c-Jun N-terminal kinase
LPS: lipopolysaccharide
MAPKs: Mitogen-activated protein kinases
M-CSF: Macrophage colony-stimulating factor
NFATc1: Nuclear factor of activated T cells c1
NFκB: Nuclear factor kappa-B
PGE_2_: Prostaglandin E_2_
RANKL: Receptor activator of nuclear factor kappa-B ligand
Tb.N: Trabecular number
Tb.Sp: Trabecular separation
Tb.Th: Trabecular thickness
TRAF6: Tumor necrosis factor receptor-associated factor6
TRAP^+^ MNCs: TRAP-positive multinucleated cells
Vitamin D_3_: Dihydroxyvitamin D_3_
XTT: Sodium 3ʹ-[1-(phenyl-aminocarbonyl)-3,4-tetrazolium]-bis(4-methoxy-6-nitro)
α-MEM: α-minimum essential medium
